# Associations between physical activity and cardiorespiratory fitness and adverse outcomes in patients with atrial fibrillation: a prospective cohort study

**DOI:** 10.3389/fcvm.2025.1570026

**Published:** 2025-04-07

**Authors:** Yanwen Chen, Yutong Wang, Xinyang Song, Tao Xu, Fang Wang

**Affiliations:** ^1^ Cardiology Department, Beijing Hospital, National Center of Gerontology, Institute of Geriatric Medicine, Chinese Academy of Medical Sciences & Peking Union Medical College, Beijing, China; ^2^ Cardiology Department, Beijing Hospital, Peking University Fifth School of Clinical Medicine, Beijing, China; ^3^ Cardiology Department, Beijing Hospital, National Center of Gerontology, Beijing, China

**Keywords:** physical activity, cardiorespiratory fitness, maximal oxygen uptake, atrial fibrillation, adverse cardiovascular outcome

## Abstract

**Background:**

Cardiorespiratory fitness (CRF) and physical activity (PA) are crucial for health and are gaining prominence in sports cardiology and rehabilitation medicine. This research study analyzes the impact of CRF and PA on the cardiovascular prognosis of patients with atrial fibrillation (AF), offering insights to optimize exercise interventions and enhance the scientific use of exercise in health.

**Methods:**

Cox regression models were used to assess the associations between CRF, PA, and endpoint events, including heart failure, stroke, myocardial infarction, and all-cause mortality. PA was categorized into four intensity levels, while CRF was quantified using three metrics: maximal oxygen uptake, (VO_2max_) resting heart rate (RHR), and maximum heart rate. To further examine the dose–response relationship, restricted cubic spline models were employed to assess potential non-linear associations.

**Results:**

Increased total physical activity [hazard ratio (HR) = 0.978; 95% confidence interval (CI): 0.961–0.995, *P* = 0.011], moderate-to-vigorous PA (HR = 0.960; 95% CI: 0.929–0.992, *P* = 0.014), and moderate physical activity (MPA) (HR = 0.953; 95% CI: 0.918–0.990, *P* = 0.014) were significantly associated with a decreased risk of major adverse cardiovascular events, respectively. All levels of PA were significantly associated with a decreased risk of cardiovascular death with HRs ranging from 0.577 to 0.938 (*P* < 0.01). Higher RHR was significantly associated with an increased risk of adverse outcomes, but not with stroke (HR = 0.999, 95% CI: 0.991–1.007, *P* = 0.7854) or cardiovascular death (HR = 1.004, 95% CI: 0.996–1.013, *P* = 0.3504). In addition, higher VO_2max_ was significantly associated with a lower risk of adverse outcomes, except for cardiovascular death (HR = 0.956, 95% CI: 0.888–1.030, *P* = 0.2376).

**Conclusion:**

This study shows that MPA significantly reduces cardiovascular risks in patients with AF, with all PA levels lowering mortality. Any PA level is beneficial, leading to immediate improvements, but excessive PA may yield diminishing returns or risks. Focusing solely on intensity or duration is insufficient; scientifically designed interventions, especially those boosting CRF (e.g., VO_2max_), have a greater effect on AF prognosis. Future programs should integrate scientifically grounded strategies to maximize benefits.

## Introduction

Atrial fibrillation (AF), a cardiac disorder characterized by rapid and disorganized electrical conduction in the atria leading to irregular heart rhythms, affects approximately one-quarter of the global population at some point in their lives (1). The occurrence of AF is closely linked to risk factors such as obesity, hypertension, alcohol consumption, and chronic kidney disease, making patients prone to aging and multiple comorbidities (2). These factors significantly impact the quality of life and prognosis of individuals with AF, increasing their susceptibility to adverse cardiovascular events, such as heart failure (HF), stroke, and death (3).

In modern society, as economies develop, there has been an increasing emphasis on healthy lifestyles and their potential benefits, with a particular focus on physical activity (PA) (4–6). Many studies have demonstrated the effects of physical activity on disease and its outcomes (7, 8). The World Health Organization (WHO) and European Society of Cardiology (ESC) recommend that all adults engage in 150–300 min of moderate physical activity (MPA), 75–150 min of vigorous physical activity (VPA), or an equivalent combination of MPA and VPA aerobic physical activity each week (6). The American College of Cardiology (ACC) also recommends 75 min or more of VPA or 150 min or more of MPA per week to reduce cardiovascular disease risk (9). The 2024 ESC guideline for the management of AF stated that moderate aerobic exercise reduces the risk of new-onset AF, but athletes appear to have a higher risk (10).

Notably, individuals can adjust their physical activity levels at any time within their capabilities, and different levels can have distinct effects (11, 12). Unlike PA, which can be adjusted at any time within an individual's capabilities, cardiorespiratory fitness (CRF) (13, 14) is a relatively stable indicator of aerobic capacity and circulatory system efficiency, serving as a reliable measure of fitness and overall health (15–19). Despite its importance, CRF levels have declined globally, attributed to factors such as sedentary behavior, obesity, socioeconomic changes, and decreasing physical activity levels (20).

In this study, besides maximal oxygen uptake (VO_2max_), which directly reflects cardiorespiratory fitness, we also include two other important indicators of CRF, resting heart rate (RHR) and maximum heart rate (HR_max_). RHR, regulated by the autonomic nervous system, is a predictor of mortality and prognosis in many cardiovascular diseases and of the occurrence of cardiovascular disease in healthy individuals (21–23). HR_max_ indicates cardiac pacemaker function and is an independent risk factor associated with aging in humans (24).

In summary, physical activity and cardiorespiratory fitness are two central concepts with significant clinical relevance in the pathogenesis and prognosis of cardiovascular diseases. There have been several advances in research on the effects of PA on AF (25–29), and we integrated PA and CRF to comprehensively evaluate their impact on cardiovascular outcomes in patients with AF. We assessed the independent predictive value of PA and CRF for adverse cardiovascular outcomes in this population. Through this investigation, we aim to provide evidence for more precise cardiovascular risk management in patients with AF and establish a theoretical foundation for the development of personalized exercise prescriptions.

## Methods

### Data source and study participants

The UK Biobank is a prospective cohort study based on a large sample of people, with over 500,000 adults aged 37–72 years recruited from 2006 to 2010 at 22 assessment centers in Wales, England, and Scotland. All participants provided informed consent before participation and this study was ethically reviewed by the North West Multi-center Research Ethics Committee (reference 11/NW/0382). Due to the sensitivity and confidentiality of the data, more detailed requests for access to the data can be sent to the UK Biobank (https://www.ukbiobank.ac.uk/), where qualified researchers will be granted access rights (30). This study includes various data from the UK Biobank, including objective device measurement of PA data, medical history and records of participants, and touchscreen questionnaires. The inclusion of the atrial fibrillation population in this study was based on the ICD10 diagnostic codes from the UK Biobank.

**Figure 1:**
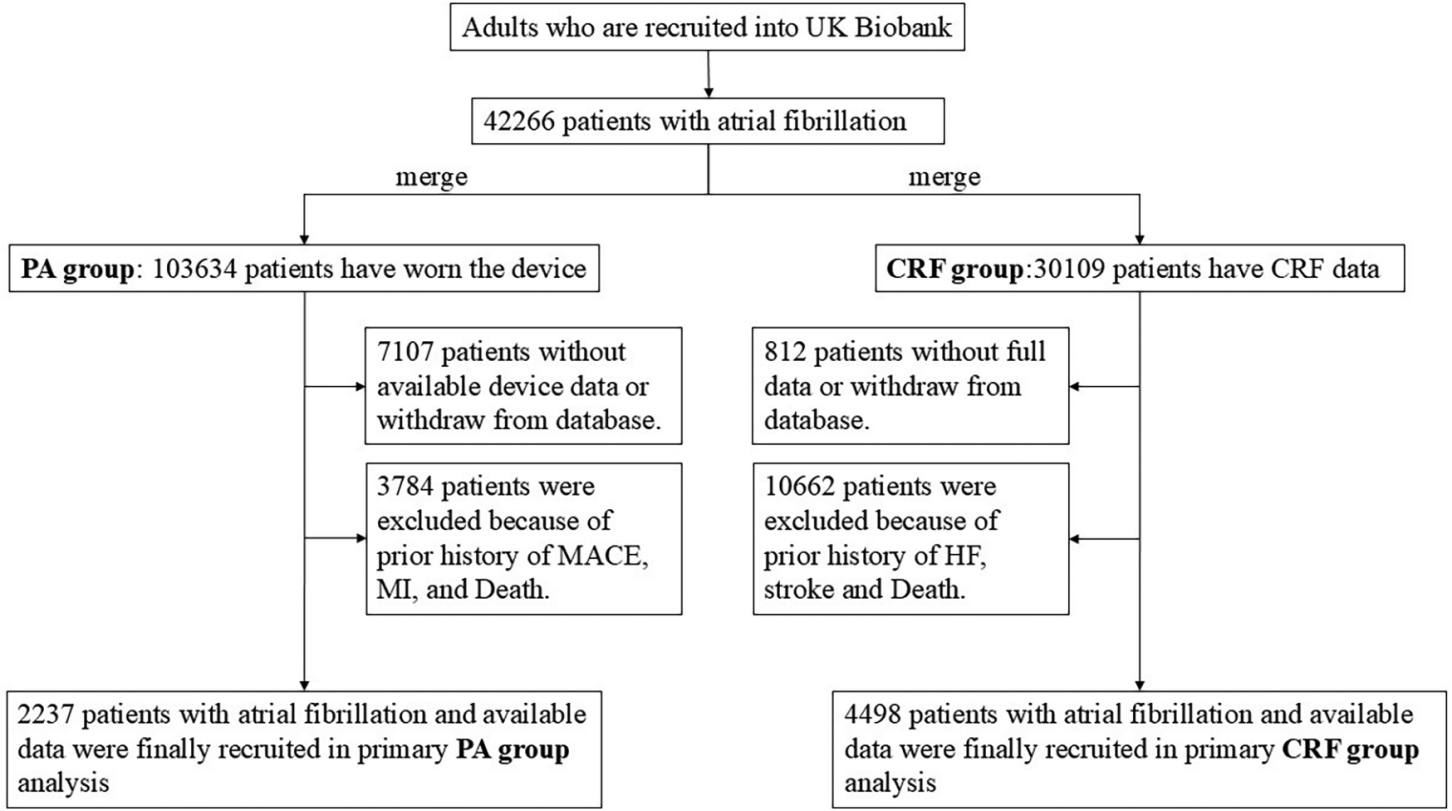
Flowchart of population screening in this study. PA, physical activity; CRF, cardiorespiratory fitness.

In this study, due to the significant time gap of several years between the date of wrist-worn accelerometer device usage and the baseline data collection, the population was divided into two independent groups to enhance the accuracy of the results. The analysis of the first group, referred to as the PA group, utilized an accelerometer device to measure PA, while the analysis of the second group, the CRF group, did not involve PA data and focused solely on baseline measurements of CRF. These two groups were mutually independent and represented distinct components of the study. The flowchart for the population screening is shown in Figure 1.

For the CRF group, we excluded individuals who (1) had withdrawn consent for participation in the UK Biobank, (2) lacked cardiorespiratory fitness data, or (3) had a history of severe outcomes at baseline or were deceased. A total of 4,498 participants with AF were included in the analysis.

For the PA group, participants were recruited from the wrist-worn accelerometer project and had been diagnosed with AF before they started wearing the accelerometer device. Participants with insufficient wear time (total wear time less than 72 h or the unavailable data for any 1 h within 24 h) or poor device calibration or who withdrew from the UK Biobank were excluded. In addition, participants who had been recorded with severe outcomes or who were dead at baseline were further excluded. A total of 2,237 participants with AF were included in the analysis.

### Assessment of physical activity

The UK Biobank sent out random emails inviting participants in the database to wear a wrist-worn accelerometer for 7 days between February 2013 and December 2015 to take measurements and calculate physical activity. The participants who agreed to this invitation received an Axivity AX3 wrist-worn triaxial accelerometer from June 2013, designed by Newcastle University Open Labs. The accelerometer device collected seven consecutive days of triaxial acceleration data with a sampling frequency of 100 Hz and a dynamic range of ±8 g. More information on data acquisition and processing is provided on the UK Biobank website (https://biobank.ndph.ox.ac.uk/ukb/label.cgi?id=1008) (31). This device records data in milligravity units. According to the convention, the three levels of physical activity, i.e., light physical activity (LPA), MPA, and VPA, were defined as 30–125, 125–400, and >400 mg, respectively (32, 33). According to the conversion formula, PA of different intensities was converted to metabolic equivalent of task (MET) minutes per week. MET was calculated as VPA × 8, MPA × 4, and LPA × 2. In addition, total physical activity (TPA) was the sum of VPA, MPA, and LPA in MET minutes per week, and the sum of MPA and VPA was moderate-to-vigorous PA (MVPA).

### Assessment of cardiorespiratory fitness

The submaximal bicycle ergometer test was conducted using a stationary eBike equipped with firmware v1.7. Participants were assigned individualized exercise protocols, and electrocardiograms (ECGs) were recorded (CAM-USB 6.5; Cardiosoft software version 6.51) across four distinct phases: the pretest phase (15 s), the constant phase (2 min at a workload of 30 W for women and 40 W for men), the incremental phase (4 min with workload progression to 35% of the maximum estimated workload for low-risk participants and 50% for minimal-risk participants), and the recovery phase (1 min).

Maximum workload was estimated using a predictive model incorporating age and physical examination findings. It is important to note that in the UK Biobank, ergometry was restricted to a maximum of 50% of the intended maximum workload. Resting heart rate was measured by ECG during a period of quiet rest. Estimated VO_2max_ was calculated using a previously validated method (34, 35), with data expressed in METs, where 1 MET corresponds to 3.5 ml/kg/min (36).

### Assessment of outcomes

The outcomes of the study include HF, stroke, myocardial infarction (MI), all-cause death, cardiovascular death, and major adverse cardiovascular event (MACE), which is defined as a composite of cardiovascular death, stroke, and MI. In the UK Biobank, the death registration information was provided by the National Death Register. Outcomes were defined according to the three-character codes from the International Classification of Diseases, tenth edition (ICD-10). The end of follow-up was set as the date of outcome diagnosis, lost to the follow-up, death, or censoring date, whichever came first. The period between the start date and the end date was defined as the follow-up time.

### Assessment of covariates

The study adjusted for several potential confounders, including demographic characteristics, lifestyle habits, physical examination results, and self-reported medication history. Demographic characteristics, such as sex (female or male), ethnicity (white population or other population), and education level (college/university degree or others), were obtained at baseline through touchscreen questionnaires. In particular, the age of the PA group was assessed at the time of wearing the accelerometer device. Socioeconomic status was defined using the Townsend deprivation index, an indicator reflecting the economic level of each participant. Lifestyle habits, including smoking status and alcohol consumption, were categorized into three levels based on self-reports: never, former, and current use. In addition, dietary habits were assessed using a 24-h recall of various foods such as fruits, vegetables, and meat, and were classified into three levels, i.e., favorable, moderate, and unfavorable, based on dietary quality assessments from a previous study (37). The self-reported medication history included whether participants had previously taken antihypertensive drugs, cholesterol-lowering drugs, or insulin.

### Statistical analysis

In this study, continuous variables were expressed as means and standard deviations, and categorical variables were expressed as frequencies and percentages. The baseline demographic and medical characteristics data of the PA group were presented in four groups according to metabolic equivalent quartiles of TPA. The baseline demographic and medical characteristics data of the CRF group were presented in two groups according to gender. Statistical methods employed for the analysis of intergroup differences included one-way analysis of variance (ANOVA) and Chi-square tests. First, the Cox proportional hazard model was used to investigate the association between exposure and the risk of outcomes. PA was considered a categorized variable, which was categorized into four groups by quartiles, and a continuous variable. When considered a continuous variable, PA was converted to MET minutes per week (LPA × 2, MPA × 4, and VPA × 8) and 1 unit PA was defined as 200 min of MET per week (100 min of LPA, 50 min of MPA, or 25 min of VPA per week) (12). Next, the dose–response relationship between different intensities of PA and CRF and adverse outcomes was investigated using a restricted cubic spline (RCS) model, with nodes selected based on model fit. All of the above analyses were adjusted with the covariates described above. A two-sided *P*-value <0.05 was considered statistically significant. All analyses and data visualization in this study were performed using R software (version 4.2.1).

## Results

### Baseline characteristics of participants

In the PA group, a total of 2,237 participants with 1,463 (73.45%) men and a mean age of 67.38 (SD 6.22) years were included in our primary analysis. After a median of 8.56 (Q1 7.98, Q4 9.14) years of follow-up, 240 MACEs, 84 cardiovascular death events, and 110 MI events were recorded. Descriptive characteristics at baseline according to the TPA quartiles are presented in Supplementary Table S1. Supplementary Table S2 presents the baseline characteristics of the CRF group. In the PA group, a total of 4,498 participants with 3,105 (69.03%) men were included. The average age was 62.74 years for men and 62.41 years for women. The VO_2max_ values of the women were lower than those of the men.

### Associations between different intensities of PA and risk of cardiovascular adverse outcomes in an AF population

**Figure 2:**
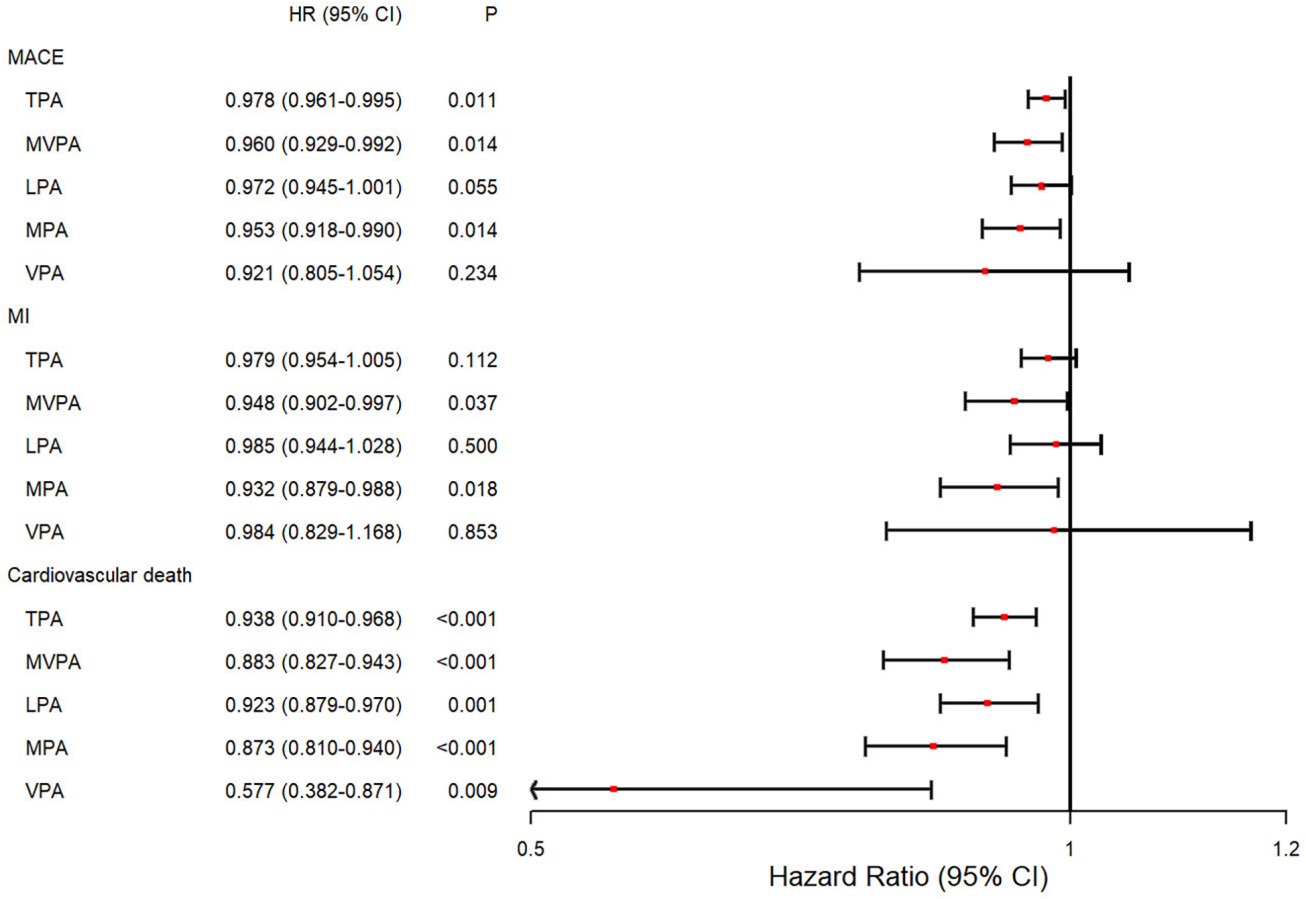
Forest plot of the associations between different intensities of PA and the risk of incident outcomes. Cox proportional hazard model adjusted for age at the time of wearing the accelerometer device, sex, ethnicity, education level, Townsend deprivation index stage, alcohol assumption status, smoking status, diet quality level, history of cardiovascular disease, and self-report medication intake, including cholesterol-lowering drugs, blood pressure, or insulin. HR, hazard ratio; CI, confidence interval; TPA, total physical activity; MVPA, moderate-to-vigorous physical activity; LPA, light physical activity; MPA, moderate physical activity; VPA, vigorous physical activity.

First, PA at each intensity was considered as a continuous variable in the longitudinal analysis (Figure 2). After adjusting for covariates, increased TPA [hazard ratio (HR) = 0.978; 95% confidence interval (CI): 0.961–0.995, *P* = 0.011], MVPA (HR = 0.960; 95% CI: 0.929–0.992, *P* = 0.014), and MPA (HR = 0.953; 95% CI: 0.918–0.990, *P* = 0.014) were significantly associated with a decreased risk of MACE events, respectively. Furthermore, MVPA (HR = 0.948; 95% CI: 0.902–0.997, *P* = 0.037) and MPA (HR = 0.932; 95% CI: 0.879–0.988, *P* = 0.018) were associated with a reduced risk of MI. Notably, all PA intensities were significantly associated with a decreased risk of cardiovascular death with HRs ranging from 0.577 to 0.938 (*P* < 0.01).

**Figure 3:**
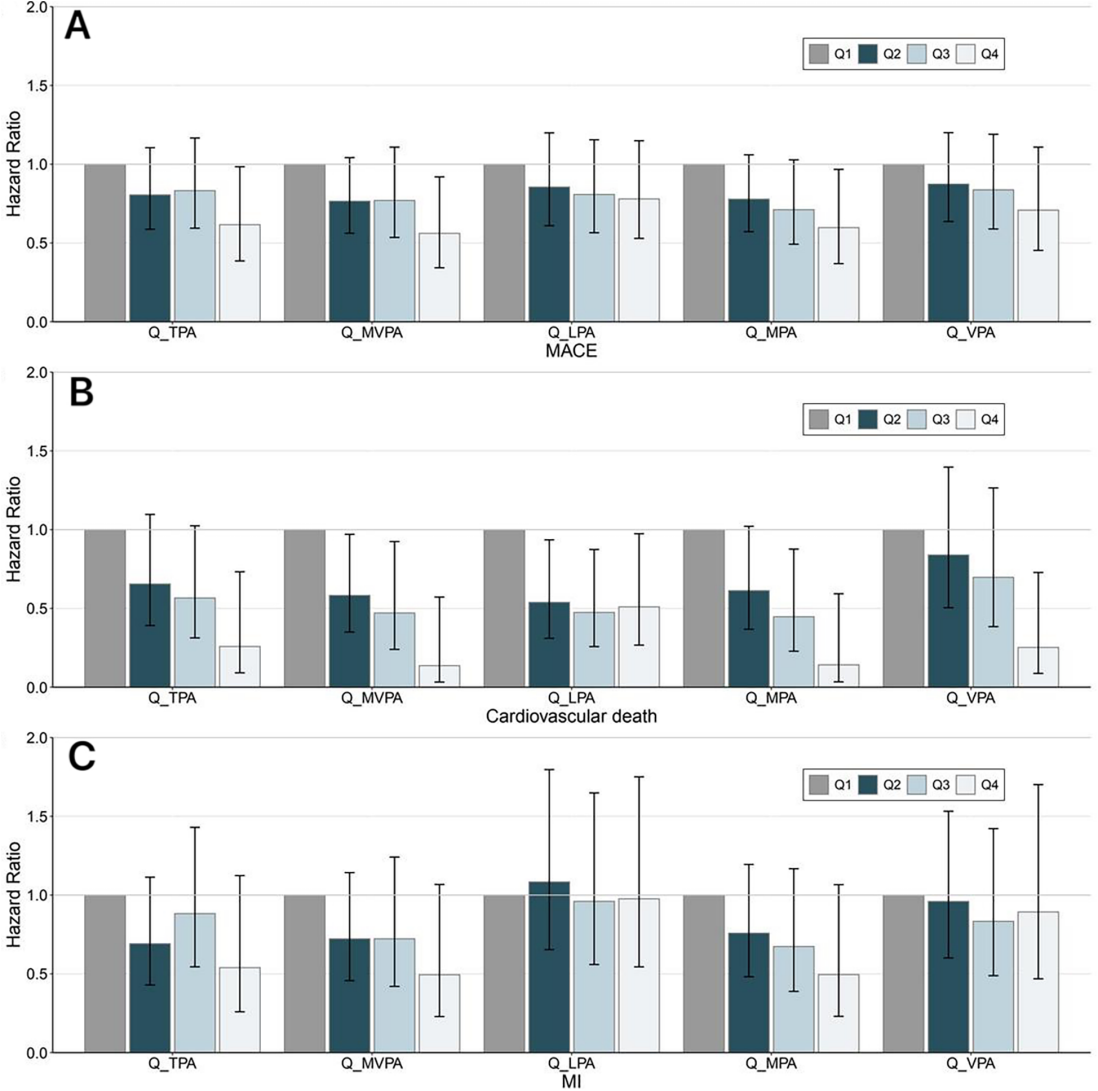
Trend of the association between PA and cardiovascular adverse outcomes. Different intensities of PA were stratified by the quartiles of PA volume, with Q1 as the reference group. Panel **(A–C)** represent MACE, cardiovascular death, and MI, respectively. The Cox regression adjusted for age, sex, ethnicity, education, Townsend deprivation index, smoking status, alcohol consumption status, diet quality level, blood pressure, history of cardiovascular disease, and self-report medication intake, including cholesterol-lowering drugs, blood pressure, or insulin. The general association trends between PA and the outcomes were tested and *P* < 0.05 was deemed statistically significant. MACE, major adverse cardiovascular events; MI, myocardial infarction; TPA, total physical activity; MVPA, moderate-to-vigorous-intensity physical activity; LPA, light-intensity physical activity; MPA, moderate-intensity physical activity; VPA, vigorous-intensity physical activity; Q, quartile.

Next, PA was further stratified by quartiles into four groups (Q1, Q2, Q3, and Q4), with Q1 as the reference group (Figure 3). Compared with the Q1 group, the TPA (HR = 0.616; 95% CI: 0.386–0.984, *P* = 0.043), MVPA (HR = 0.561; 95% CI: 0.343–0.920, *P* = 0.022), and MPA (HR = 0.598; 95% CI: 0.369–0.967, *P* = 0.036) of the Q4 group (highest PA group) were significantly associated with a decreased risk of incident MACEs (Figure 3A). There were no significant associations between the different PA quartile groups and the risk of incident MI (Figure 3C). Moreover, when analyzing cardiovascular mortality as the endpoint, individuals in the higher PA quartiles demonstrated a reduced risk of incident cardiovascular death compared to the Q1 group (Figure 3B). Conversely, the LPA groups exhibited no significant differences in HR across quartiles Q2 (HR = 0.539; 95% CI: 0.310–0.935, *P* = 0.028), Q3 (HR = 0.475; 95% CI: 0.258–0.874, *P* = 0.017), and Q4 (HR = 0.510; 95% CI: 0.267–0.974, *P* = 0.041).

### Association between CRF and the risk of cardiovascular adverse outcomes in an AF population

Table 1 summarizes the effects of cardiorespiratory fitness and heart rate on adverse cardiovascular outcomes in AF populations. A higher RHR was significantly associated with an increased risk of adverse outcomes, specifically HF (HR = 1.007, 95% CI: 1.002–1.012, *P* = 0.0047) and all-cause death (HR = 1.009, 95% CI: 1.004–1.014, *P* < 0.0001), but not with stroke (HR = 0.999, 95% CI: 0.991–1.007, *P* = 0.7854) or cardiovascular death (HR = 1.004, 95% CI: 0.996–1.013, *P* = 0.3504). In addition, higher VO_2max_ was significantly associated with a lower risk of adverse outcomes, including HF (HR = 0.934, 95% CI: 0.899–0.972, *P* < 0.0001), stroke (HR = 0.943, 95% CI: 0.891–0.999, *P* = 0.0446), and all-cause death (HR = 0.957, 95% CI: 0.918–0.998, *P* = 0.038), except for cardiovascular death (HR = 0.956, 95% CI 0.888–1.030, *P* = 0.2376). In patients with AF, HR_max_ was not associated with adverse cardiovascular outcomes.

**Table 1:** Hazard ratios for the CRF group and all the outcomes.

Outcome	Resting heart rate		Maximum heart rate		VO_2max_	
	HR (95% CI)	*P*-value	HR (95% CI)	*P-*value	HR (95% CI)	*P*-value
HF	1.007 (1.002–1.012)	0.0047	0.999 (0.992–1.005)	**0.6879**	0.934 (0.899–0.972)	<0.001
Stroke	0.999 (0.991–1.007)	**0.7856**	0.995 (0.984–1.006)	**0.3396**	0.943 (0.891–0.999)	0.0446
All-cause death	1.009 (1.004–1.014)	<0.0001	1.003 (0.996–1.010)	**0.3912**	0.957 (0.918–0.998)	0.038
Cardiovascular death	1.004 (0.996–1.013)	**0.3504**	0.992 (0.978–1.005)	**0.2292**	0.956 (0.888–1.030)	**0.2374**

AF, atrial fibrillation; HF, heart failure; VO_2max_, maximal oxygen consumption.

The Cox proportional hazard models were adjusted for age, sex, ethnicity, education, Townsend deprivation index, smoking status, alcohol consumption, diet quality, blood pressure, waist-to-hip ratio, body mass index, parental history of cardiovascular disease, and personal history of antihypertensive, lipid-lowering, or glucose-lowering medication use. The bold values represent that *P*-values are meaningless.

### Dose–response association between the exposures and cardiovascular adverse outcomes in an AF population

**Figure 4:**
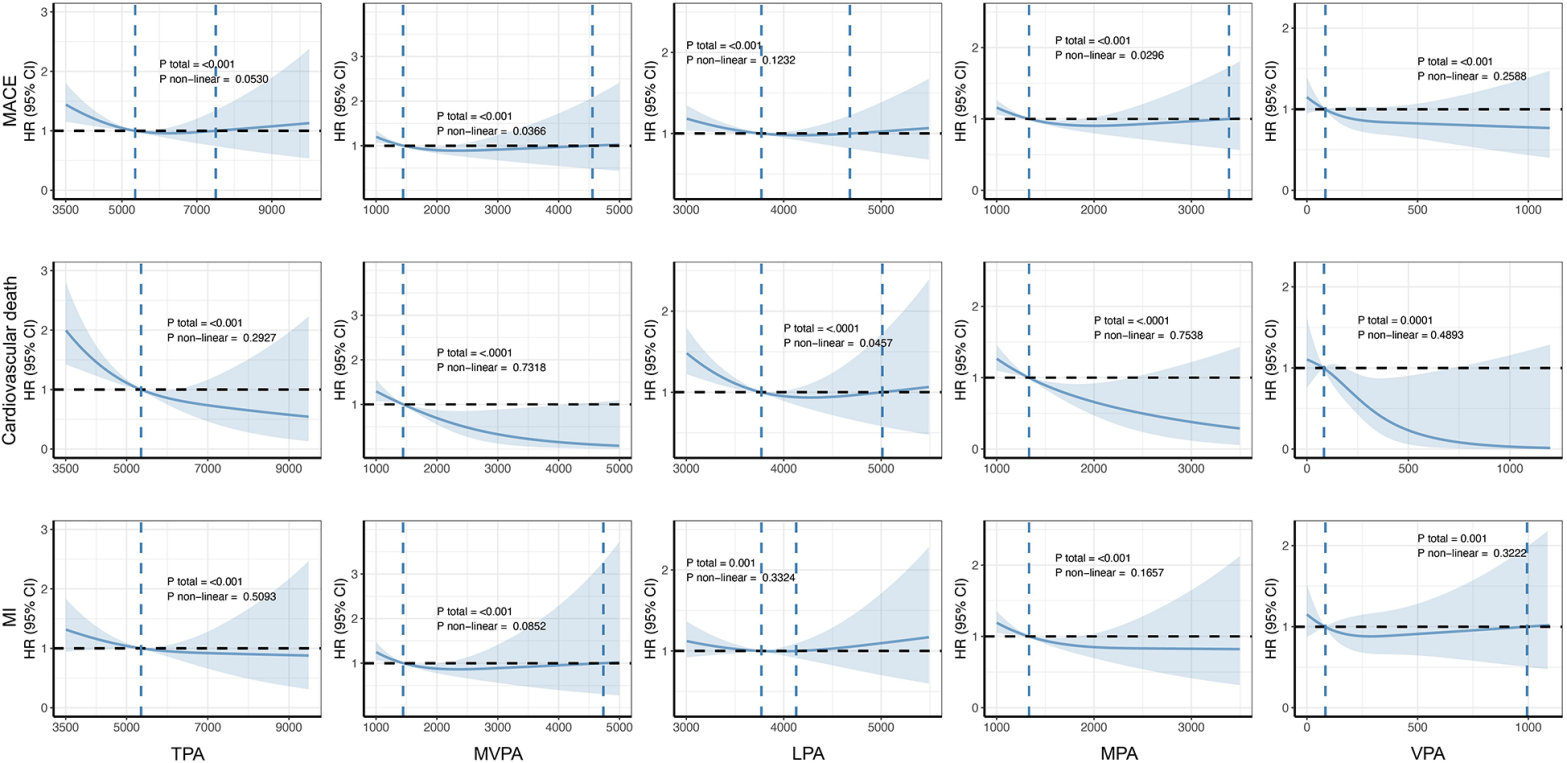
Dose–response associations between PA and cardiovascular adverse outcomes. The Cox regression models with restricted cubic splines were used to explore the dose–response relationship between PA and the outcomes. The HRs and their 95% CIs were calculated. All were adjusted for age, sex, ethnicity, education, Townsend deprivation index, smoking status, alcohol consumption status, diet quality level, blood pressure, history of cardiovascular disease, and self-report medication intake, including cholesterol-lowering drugs, blood pressure, or insulin. MACE, major adverse cardiovascular event; MI, myocardial infarction; TPA, total physical activity; MVPA, moderate-to-vigorous-intensity physical activity; LPA, light-intensity physical activity; MPA, moderate-intensity physical activity; VPA, vigorous-intensity physical activity.

**Figure 5:**
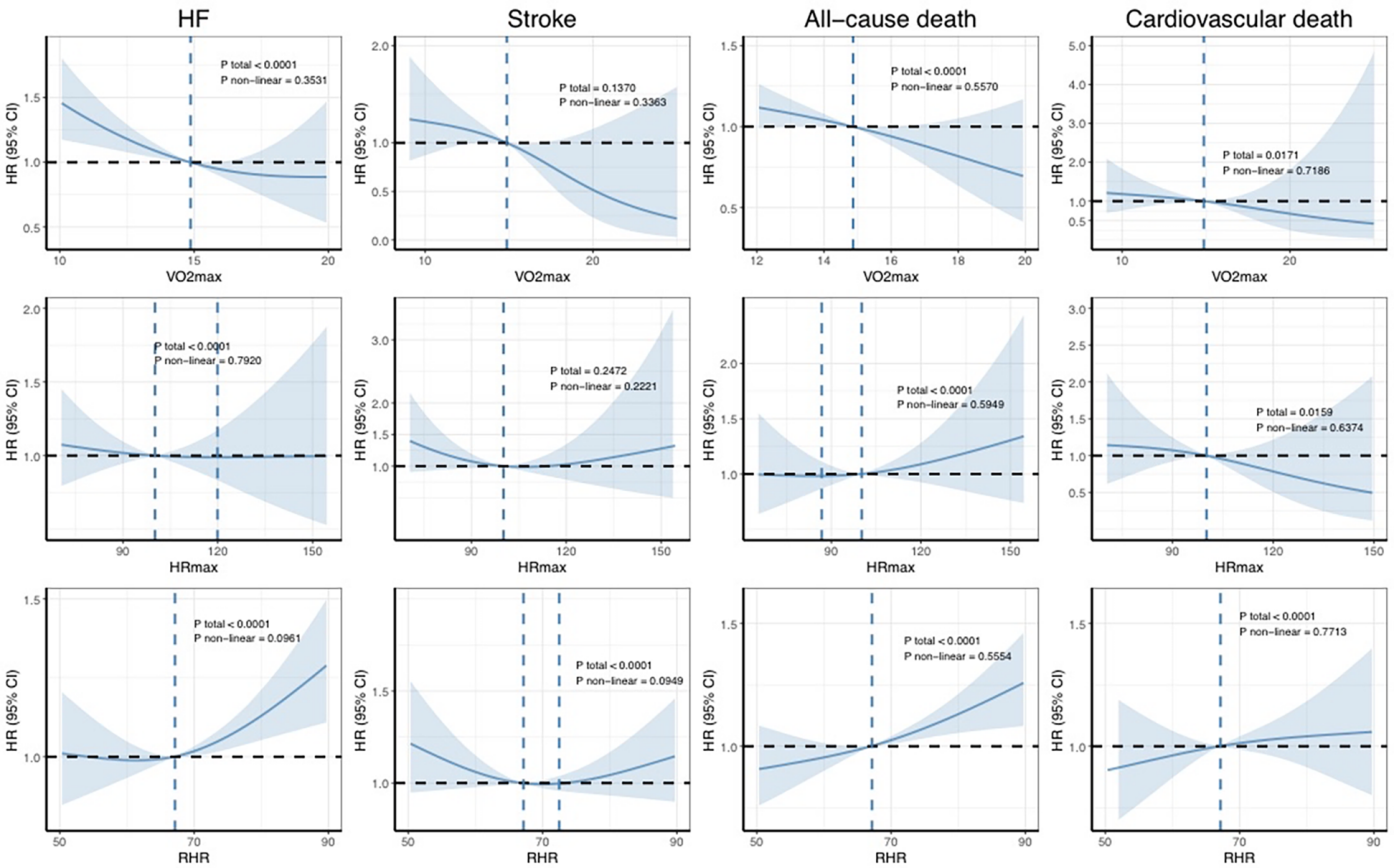
Dose–response associations between CRF and the risk of incident outcomes. The Cox regression models with restricted cubic splines were used to explore the dose–response relationship between cardiorespiratory fitness and the outcomes. The HRs and their 95% CIs are shown. The Cox proportional hazard models were adjusted for age, sex, ethnicity, education, Townsend deprivation index, smoking status, alcohol consumption, diet quality, blood pressure, waist-to-hip ratio, body mass index, parental history of cardiovascular disease, and personal history of antihypertensive, lipid-lowering, or glucose-lowering medication use. HR, hazard ratio; CI, confidence interval; AF, atrial fibrillation; HF, heart failure.

Figure 4 shows the dose–response association between PA and the occurrence of outcomes in patients with AF, as calculated using the RCS model. Overall, consistent with previous findings, a greater volume of PA at varying intensities was associated with a reduced risk of adverse cardiovascular outcomes. Notably, only the associations between MVPA and MACE and between LPA and cardiovascular death were statistically significant non-linear associations (*P*_non-linear_ = 0.0366 and 0.0457, respectively). In contrast, no significant non-linear relationships between CRF and severe outcomes were observed in the AF population (Figure 5).

## Discussion

The main findings of this study are as follows. First, PA of all intensities was associated with a reduction in cardiovascular mortality in patients with AF, with moderate-intensity PA linked to a lower incidence of MI. When PA levels were categorized by quartiles, with Q1 as the reference group, higher quartile levels of PA were associated with a reduced risk of cardiovascular mortality. Second, a higher resting heart rate was associated with an increased risk of death, while higher VO_2max_ was significantly associated with a lower risk for all outcomes. Finally, most of the results in this study did not exhibit non-linear relationships but instead showed linear associations.

Next, we will provide a detailed analysis of the results. First, the analysis of the association between PA intensity and the risk of a MACE revealed that only MPA (e.g., brisk walking and recreational cycling) and MVPA (e.g., jogging and swimming) were significantly associated with a reduced incidence of a MACE. In contrast, LPA (e.g., casual walking and routine household chores) did not provide significant cardiovascular protective effects, likely due to insufficient exercise load. Similarly, while VPA (e.g., competitive sports) demonstrated potential benefits in enhancing cardiorespiratory fitness, its associated cardiovascular stress may have offset its protective effects. A similar trend was observed in the clinical outcome of MI. Notably, however, PA at all intensity levels was significantly associated with reduced mortality in patients with AF, demonstrating an independent protective effect on survival. This finding suggests that while the impact of different PA intensities may vary for specific cardiovascular outcomes, the overall benefit of PA in reducing mortality among patients with AF is universal. These effects are likely mediated through mechanisms such as improved overall cardiovascular health, enhanced physiological function, and optimized metabolic status. Different intensities of physical activity confer substantial metabolic benefits in patients with AF by improving insulin sensitivity, reducing adiposity, and enhancing lipid profiles, which collectively diminish systemic inflammation and adverse cardiac remodeling. Regular physical activity increases high-density lipoprotein (HDL) cholesterol and also reduces triglycerides and small, dense low-density lipoprotein (LDL) particles, which may decrease the atherosclerotic burden and endothelial dysfunction.

Second, when the cumulative time spent in physical activity was stratified into quartiles, the analysis revealed that increased exercise duration does not always correlate linearly with cardiovascular protective effects. For example, the incidence of MACEs in the third quartile was higher than in the second quartile of MVPA. This finding suggests that excessively prolonged MVPA at certain intensities may not further improve cardiovascular outcomes and could even lead to adverse effects, such as increased oxidative stress or cardiovascular overload, associated with overexercise. These results underscore the importance of balancing exercise intensity and duration, highlighting that excessive exercise interventions may be counterproductive.

Nevertheless, it is noteworthy that regardless of intensity, participants engaging in any amount of physical activity above the first quartile had significantly lower adverse outcome rates compared to the completely sedentary control group (first quartile). This finding reinforces the public health message that “any exercise is better than none” and emphasizes the need to carefully balance intensity and duration when designing personalized exercise prescriptions to achieve optimal cardiovascular benefits.

Unlike PA, which is activity-dependent, CRF is an inherent physiological attribute that does not rely on the intensity or duration of a single exercise session. Instead, CRF can be enhanced through long-term, systematic, and scientifically guided exercise training. Our study demonstrated that higher CRF levels were independently associated with a significantly lower incidence of HF and stroke. This finding highlights the importance of prioritizing systematic planning at a macro level when designing exercise rehabilitation programs, focusing on improving CRF through structured and scientifically informed interventions rather than narrowly emphasizing exercise intensity or duration. Furthermore, the results underscore that unstructured or arbitrary exercise regimens may fail to effectively improve CRF and may not deliver the anticipated cardiovascular benefits due to a lack of specificity. Future strategies for exercise rehabilitation should emphasize individualized, long-term planning that incorporates various modalities. These comprehensive approaches aim to enhance CRF, thereby reducing the risk of adverse cardiovascular events. CRF may enhance cardiac efficiency in patients with AF through improved cardiac output and ventricular function, creating a more favorable hemodynamic profile with reduced wall stress and filling pressures. Furthermore, higher CRF levels are consistently associated with lower inflammatory biomarkers including C reactive protein or interleukin 6 (IL-6), which directly contribute to adverse atrial electrical and structural remodeling. Better CRF may promote favorable sympathetic balance, potentially reducing the arrhythmia burden and improving heart rate control. These mechanisms provide powerful physiological explanations for the reductions in adverse outcomes in patients with AF.

Analysis of the RCS curves for PA and AF and for CRF and AF reveals one difference. With increasing PA, the curve gradually stabilizes and even shows a slight upward trend. This pattern suggests that while accumulating PA offers benefits, these gains may diminish beyond a certain threshold, potentially even reversing. In contrast, the RCS curve for VO_2max_ and AF demonstrates a consistently linear downward trajectory. This finding indicates that improvements in VO_2max_ are unlikely to encounter a similar plateau effect or exhibit a distinct “inflection point.” In non-professional fitness regimens, individuals typically do not undergo cardiopulmonary exercise testing (CPET) to measure their VO_2max_, nor do they routinely monitor their heart rates during physical activity or in resting states. In contrast, the HUNT study, a comprehensive, prospective cohort investigation in Norway, has diligently focused on evaluating and augmenting cardiopulmonary function in the general populace (38–43). By improving cardiopulmonary fitness, the study aims to optimize exercise performance and ultimately enhance cardiovascular outcomes. The approach and findings of this research have potential implications for public health interventions and applications.

In this study, two mutually independent populations of patients with AF were analyzed, rather than a single cohort. This approach was necessitated by limitations in the data format of the UK Biobank database. The UK Biobank, a prospective cohort study initiated in 2006, spans a prolonged timeline, with accelerometer technology introduced only after 2013. This extended timeframe introduced potential biases due to changes over time. To mitigate these biases, we separated the accelerometer device cohort and the baseline-recruited cohort into two distinct and independent study populations. While this methodology addressed the temporal limitations, it also introduced certain drawbacks, such as the inability to examine interactions between CRF and PA within the same population. Future large-scale prospective cohort studies are needed to explore these interactions in greater depth.

This study has several other limitations. First, due to the inconvenience and difficulty of CPET (44), it is not a routine clinical examination. In this study, because of the limitations of the available data, VO_2max_ was estimated using formulas (34, 35), which may introduce some inaccuracies. However, the data used for the calculations—heart rate, age, and weight—were directly measured instead of estimated, enhancing the credibility and reliability of the results.

Second, the scale of the study population was not large enough and the proportions of white participants and middle-aged and elderly individuals were relatively high, leading to a skewed distribution of data such as heart rate, which may introduce bias.

Third, in this study, physical activity data were collected for 7 days, which may not fully reflect long-term exercise habits. Long-term, sustained physical activity produces adaptive changes in cardiac structure, and the effects of physical activity on cardiac structure and human metabolism are long-lasting (45). In addition, variations in exercise effects may arise due to differences in seasons, days of the week, or times of the day. Hence, further long-term and continuous studies are needed to validate these findings.

Finally, since AF can affect heart rate, further studies in patients with other cardiovascular diseases are needed in the future. Future studies should focus on more targeted investigations in patients with AF and in those with concomitant cardiovascular diseases.

With the advancement of multidisciplinary integration and the widespread application of the “exercise is medicine” concept in clinical practice, the intersection of cardiac rehabilitation and sports medicine has grown increasingly prominent. This offers novel theoretical and practical paradigms for the prevention and management of cardiovascular diseases. In this context, our study examined the effects of physical activity at varying intensities (low, moderate, and high) and durations on adverse cardiovascular outcomes in patients with AF. In addition, we innovatively incorporated cardiorespiratory fitness as a key exposure variable to comprehensively evaluate the combined impact of PA and CRF on prognosis in this population.

The findings of our study highlight that while moderate and appropriately dosed exercise interventions are essential for improving cardiovascular outcomes, enhancing individual CRF levels is equally critical and may even be a pivotal factor in optimizing prognosis. Evidence suggests that scientifically guided exercise interventions—individualized aerobic training, high-intensity interval training (HIIT), and resistance training—can significantly improve CRF, thereby amplifying their cardiovascular benefits. Conversely, unsupervised or improperly executed exercise regimens may fail to deliver the desired benefits and, in some cases, increase the risk of adverse cardiovascular events due to overexertion or inappropriate exercise methods.

In conclusion, this study provides novel evidence to guide cardiovascular risk management in patients with AF and underscores the central role of scientifically designed exercise interventions in improving CRF and prognosis. Future research should focus on improving individualized exercise prescription strategies through multidisciplinary collaboration, thereby offering more precise and effective approaches for the prevention and rehabilitation of cardiovascular diseases. This research direction not only holds significant academic value but also provides a practical pathway to improving global health.

## Data Availability

The datasets presented in this study can be found in online repositories. The names of the repository/repositories and accession number(s) can be found below: All data in this present study is available in the UK Biobank (https://www.ukbiobank.ac.uk). All data can be viewed online (https://biobank.ndph.ox.ac.uk/showcase/index.cgi), while the use of the data requires an application. The present study was approved by the UK Biobank under application number 144894 (https://ams.ukbiobank.ac.uk/ams/resMessages).
