# Supplementary material for: Associations between physical activity and cardiorespiratory fitness and adverse outcomes in patients with atrial fibrillation: a prospective cohort study
Source: Front Cardiovasc Med. 2025 Apr 7;12:1570026. doi: 10.3389/fcvm.2025.1570026 (PMC12009929; doi:10.3389/fcvm.2025.1570026)
Supplement: Supplementary file 1 [file Table1.pdf]

Supplementary Table 1. Baseline characteristics of PA group by quartiles into four groups (Q1, Q2, Q3, and Q4)

Characteristics	TPA strata				*P* value
	Q1	Q2	Q3	Q4	
Sample size, n	684	653	562	338	
Age, years, mean (SD)	68.25 (6.01)	68.00 (5.94)	66.97 (5.88)	65.19 (7.10)	<0.001
Male, n (%)	470 (68.71)	436 (66.77)	358 (63.70)	199 (58.88)	0.012
Ethnic, white, n (%)	674 (99.56)	643 (98.77)	555 (98.93)	330 (98.21)	0.220
College, n (%)	266 (39.47)	266 (41.24)	253 (45.59)	134 (39.88)	0.149
WHR, mean (SD)	0.92 (0.09)	0.90 (0.09)	0.88 (0.08)	0.86 (0.08)	<0.001
BMI, kg/m^2, mean (SD)	29.79 (5.90)	28.19 (4.90)	26.76 (4.06)	25.93 (3.71)	<0.001
SBP, mmHg, mean (SD)	140.53 (17.67)	141.05 (18.60)	139.11 (18.05)	137.13 (18.99)	0.007
DBP, mmHg, mean (SD)	83.15 (10.40)	83.01 (10.42)	82.17 (9.93)	80.53 (10.11)	<0.001
TDI, n (%)					0.110
Low	247 (36.11)	260 (39.82)	239 (42.68)	128 (37.98)	
Intermediate	228 (33.33)	232 (35.53)	178 (31.79)	121 (35.91)	
High	209 (30.56)	161 (24.66)	143 (25.54)	88 (26.11)	
Diet quality, n (%)					0.005
Healthy	34 (4.99)	25 (3.85)	38 (6.79)	26 (7.74)	
Intermediate	519 (76.21)	520 (80.00)	443 (79.11)	272 (80.95)	
Unhealthy	128 (18.80)	105 (16.15)	79 (14.11)	38 (11.31)	
Smoking status, n (%)					0.227
Never	316 (46.47)	330 (50.77)	282 (50.27)	184 (54.60)	
Previous	324 (47.65)	285 (43.85)	255 (45.45)	134 (39.76)	
Current	40 (5.88)	35 (5.38)	24 (4.28)	19 (5.64)	
Alcohol consumption, n (%)					0.200
Never	23 (3.37)	20 (3.07)	12 (2.14)	6 (1.78)	
Previous	32 (4.69)	18 (2.76)	20 (3.57)	8 (2.37)	
Current	628 (91.95)	613 (94.16)	528 (94.29)	324 (95.86)	
Medication, n (%)					
Cholesterol Lowering	197 (28.80)	141 (21.59)	112 (19.93)	37 (10.95)	<0.001
Blood Pressure Lowering	213 (31.14)	183 (28.02)	129 (22.95)	45 (13.31)	<0.001
Insulin	7 (1.02)	6 (0.92)	1 (0.18)	1 (0.30)	0.200
PA (min/week), mean (SD)					
LPA	1403.96 (296.53)	1880.73 (224.19)	2132.60 (289.91)	2427.97 (337.68)	<0.001
MPA	181.28 (87.32)	319.81 (96.93)	450.25 (124.88)	669.54 (192.37)	<0.001
VPA	7.37 (10.87)	14.25 (15.81)	27.70 (35.53)	51.13 (57.88)	<0.001

Continuity and categorical variables are shown as mean (SD) and number (percentage), respectively.

Abbreviation: BMI, body mass index; WHR, waist-to-hip ratio; TDI, townsend deprivation index; SBP: systolic blood pressure; DBP: diastolic blood pressure; TPA, total physical activity; LPA, light intensity physical activity; MPA, moderate intensity physical activity; VPA, vigorous intensity physical activity; Q, quartile.
