# Supplementary material for: Associations between physical activity and cardiorespiratory fitness and adverse outcomes in patients with atrial fibrillation: a prospective cohort study
Source: Front Cardiovasc Med. 2025 Apr 7;12:1570026. doi: 10.3389/fcvm.2025.1570026 (PMC12009929; doi:10.3389/fcvm.2025.1570026)
Supplement: Supplementary file 2 [file Table2.pdf]

Supplementary Table 2. Baseline characteristics of CRF group by gender

Characteristics	Gender		*P*
	Famale	Male	
Sample size, n	1393	3105	
Age, years, mean (SD)	62.21 (5.92)	62.09 (6.14)	0.54
Ethnic, white, n (%)	1342 (97.18)	2997 (97.12)	0.99
College, n (%)	298 (22.06)	838 (27.61)	<0.05
WHR, mean (SD)	0.83 (0.07)	0.95 (0.07)	<0.05
BMI, kg/m2, mean (SD)	28.85 (5.95)	29.03 (4.79)	0.34
SBP, mmHg, mean (SD)	138.40 (20.38)	139.22 (18.68)	0.2
DBP, mmHg, mean (SD)	79.91 (11.14)	82.86 (10.99)	<0.05
TDI, n (%)			0.5
Low	493 (35.39)	1053 (33.95)	
Intermediate	473 (33.96)	1048 (33.78)	
High	427 (30.65)	1001 (32.27)	
Diet quality, n (%)			<0.05
Healthy	103 (7.47)	136 (4.45)	
Intermediate	1126 (81.71)	2319 (75.91)	
Unhealthy	149 (10.81)	600 (19.64)	
Smoking status, n (%)			<0.05
Never	723 (52.54)	1250 (40.54)	
Previous	567 (41.21)	1584 (51.38)	
Current	86 (6.25)	249 (8.08)	
Alcohol consumption, n (%)			<0.05
Never	92 (6.65)	93 (3.01)	
Previous	114 (8.24)	168 (5.43)	
Current	1178 (85.12)	2833 (91.56)	
History of CVD, n (%)	331 (23.76)	903 (29.08)	
RHR, beats per minute, mean (SD)	64.16 (11.62)	63.60 (16.47)	0.76
HRmax, beats per minute, mean (SD)	98.97 (26.50)	101.48 (24.83)	0.24
VO2max, ml/ (kg·min), mean (SD)	12.32 (4.35)	14.84 (5.06)	<0.05

Continuity and categorical variables are shown as mean (SD) and number (percentage), respectively.

Abbreviation: BMI, body mass index; WHR, waist-to-hip ratio; TDI, townsend

deprivation index; SBP: systolic blood pressure; DBP: diastolic blood pressure; RHR: resting heart rate; HR: heart rate; VO2max: maximal oxygen consumption.
